# Prematurity, perinatal inflammatory stress, and the predisposition to develop chronic kidney disease beyond oligonephropathy

**DOI:** 10.1007/s00467-020-04712-2

**Published:** 2020-09-03

**Authors:** Lieke A. Hoogenboom, Tim G. A. M. Wolfs, Matthias C. Hütten, Carine J. Peutz-Kootstra, Michiel F. Schreuder

**Affiliations:** 1 Department of Pediatrics, Maastricht University Medical Centre+, Maastricht, The Netherlands; 2 Department of Pediatric Nephrology, Radboudumc Amalia Children’s Hospital, Nijmegen, The Netherlands; 3 Department of Pediatrics, School for Oncology and Developmental Biology (GROW), Maastricht University, Maastricht, The Netherlands; 4 Department of Biomedical Engineering (BMT), Maastricht University, Maastricht, The Netherlands; 5 Department of Neonatology, Maastricht University Medical Centre+, Maastricht, The Netherlands; 6 Department of Pathology, School for Cardiovascular Diseases (CARIM), Maastricht University Medical Centre+, Maastricht, The Netherlands

**Keywords:** Oligonephropathy, Podocytopathy, Prematurity, Intrauterine growth restriction, Chorioamnionitis

## Abstract

Prematurity and perinatal stress, such as intrauterine growth restriction (IUGR) and chorioamnionitis, are pathological processes creating an impaired intrauterine environment. These intrauterine factors are associated with the development of proteinuria, hypertension, and chronic kidney disease (CKD) later in life. Initially, this was thought to be secondary to oligonephropathy, subsequent glomerular hypertrophy, and hyperfiltration, leading to glomerulosclerosis, a further decrease in nephron number, and finally CKD. Nowadays, there is increasing evidence that prematurity and perinatal stress affect not only nephron endowment but also the maturation of podocytes and vasculogenesis. IUGR is associated with podocyte damage and an aggravated course of nephrotic syndrome. Moreover, preterm birth and IUGR are known to cause upregulation of the postnatal renin-angiotensin system, resulting in hypertension. Chorioamnionitis causes damage to the glomeruli, thereby predisposing to the development of glomerulosclerosis. This review aims to summarize current knowledge on the influence of prematurity, IUGR, and chorioamnionitis on the development of different glomerular structures. After summarizing human and experimental data on low nephron number in general, a specific focus on the current understanding of podocyte and glomerular capillary formation in relation to prematurity and different causes of perinatal stress is presented.

## Introduction

Developments in perinatal and postnatal care have increased the survival of neonates at the expense of increased morbidity later in life, with report of neurologic and pulmonary complications in up to 50% of extreme preterm or extreme low birth weight neonates. Kidney development continues until 34–36 weeks of gestation and may be hampered by preterm birth and/or perinatal stress, such as intrauterine growth restriction (IUGR) and chorioamnionitis. These constraints on kidney development are associated with hypertension, proteinuria, focal segmental glomerulosclerosis (FSGS), and chronic kidney disease (CKD) later in life. Low birth weight (LBW) is associated with a 70% increased risk and prematurity (< 28 weeks gestation) with a threefold increase in risk of CKD [1, 2]. Therefore, the increased survival of these children due to ameliorated postnatal care may lead to long-term complications and increased demands on (renal) health care.

The pathophysiological mechanisms underlying the development of proteinuria, hypertension, and CKD in the context of perinatal stress or preterm birth are not fully elucidated. Much research has been conducted into the field of reduced nephron endowment and the glomerular hyperfiltration theory as described by Brenner and colleagues in 1981, which explains how a reduced nephron number could lead to glomerular sclerosis and kidney failure [3]. However, this does not fully explain the different clinical presentations mentioned above and does not explain the increased risk of CKD later in life after late preterm or early term birth [2].

**Fig. 1:**
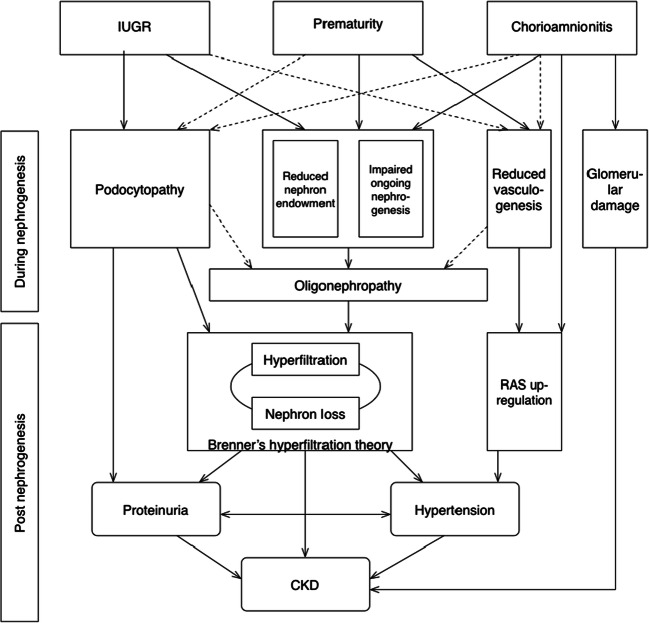
Correlations of intrauterine growth restriction (IUGR), prematurity, and chorioamnionitis with elements of glomerular development and postnatal consequences. Continuous line indicates a proven correlation; dotted line indicates a hypothesized correlation. RAS renin-angiotensin system, CKD chronic kidney disease

This raises the question of whether different pathophysiological pathways are affected by different causes of perinatal stress and the effect of their timing during gestation. There is growing evidence that in addition to the glomerular number, development of other structures such as podocytes and the vascular system are also affected. This review aims to summarize current knowledge on the influence of prematurity, IUGR, and chorioamnionitis on the development of different glomerular structures (Fig. 1). After summarizing human and experimental data on low nephron number, a specific focus on the current understanding of podocyte and glomerular capillary formation in relation to preterm birth and different causes of perinatal stress is presented.

## Renal consequences of prematurity and perinatal stress

Evidence is accumulating that prematurity and perinatal stress significantly increase the risk of postnatal kidney pathologies [4]. A meta-analysis by White et al. included 18 studies with more than 2.2 million individuals and showed that LBW was associated with a 70% greater risk of developing CKD and 60% increased risk of CKD stage 5 (CKD 5) compared with normal birth weight [5]. Such an increased risk was confirmed in a recent analysis by Das et al. [6]. However, LBW is a heterogeneous group in which only a subgroup has been exposed to the pathological process of IUGR [7]. The pathological processes, such as IUGR and chorioamnionitis, that frequently coincide with LBW are considered to further increase the risk of CKD in LBW neonates. The risk of developing CKD during childhood and adolescence is also correlated with the gestational age at birth. Data from a Swedish registry of over 4 million cases recently showed that children born extremely preterm (< 28 weeks gestation) have a three times higher risk of developing CKD before the age of 44 years. Hazard ratios (HR) for early prematurity (28–33 weeks gestation) and late prematurity (34–36 weeks gestation) were 2.2 and 1.8, respectively. Interestingly, early term birth (37–38 weeks gestation) also had a slightly increased risk (HR 1.3) of CKD [2]. Evidence regarding the effect of gender on the risk of CKD in prematurity and IUGR and its underlying pathophysiological mechanisms is growing but currently inconclusive [2, 8–10].

In the presence of comorbidities, perinatal stress and prematurity impose an additional risk of developing CKD. In an adult population of patients with hypertension and/or diabetes, Fan et al. showed that patients with LBW were 56% more likely to develop CKD than those with a normal birth weight [11]. The average age of the patients with CKD was 33.9 years, while the average age in those without CKD was 37.6 years. In patients with IgA nephropathy, Ruggajo found a significantly higher risk of developing CKD 5 in LBW (HR 2.0) and small for gestational age (SGA) (HR 2.2) patients [12]. Orskov reports on a population of patients with autosomal dominant polycystic kidney disease (ADPKD) in which birth weight correlated with the age of onset of CKD 5 [13]. Their regression analysis showed that with every kilogram decrease in birth weight, the age of onset of CKD 5 decreased by 5 years in women and 3.9 years in men. Gjerde et al. recently published registry data of patients up to the age of 50 years showing increased HR for CKD 5 in correlation with LBW (HR 1.61), SGA (HR 1.44), and prematurity (HR 1.54) [10]. The HR increased to 2.26, 1.70, and 2.32 for LBW, SGA, and prematurity, respectively, in the presence of congenital urinary tract malformations or hereditary kidney disease. Moreover, Gjerde et al. showed that a combination of LBW, SGA, and prematurity further increased the HR to 2.96 when all three are present [10]. The same pattern of progressive risk increase when combining LBW and prematurity was found in a Norwegian registry study by Ruggajo et al. [14]. Overall, the aforementioned studies confirm not only the individual effect of LBW, IUGR, and prematurity on CKD and CKD 5, but also their cumulative effects on the development of reduced kidney function.

Microalbuminuria is an early marker of glomerular hyperfiltration, and several studies report on the correlation with birth weight and prematurity. A meta-analysis by White et al. reports an 80% increased risk of developing microalbuminuria with LBW [5]. Keijzer-Veen et al. reported on a group of extreme premature patients and found that those with IUGR had a 2.4 times higher risk of microalbuminuria at the age of 19 years compared with those with birth weights appropriate for gestational age [15]. The correlation between prematurity alone and microalbuminuria is less convincing. Vashishta et al. report no difference in urinary albumin loss at the age of 30 months between prematurity (< 30 weeks) and term birth [16].

Prematurity and perinatal stress are associated with hypertension both in childhood and in adulthood. In children at the age of 30 months and born preterm (< 30 weeks), 30.9% were found to have hypertension compared with 3.9% in term born children [16]. At the age of 14 years, South et al. reported hypertension in 4% of preterm born children and 0% in those born term [17]. A follow-up study by Keijzer-Veen presented blood pressure data on children born preterm (< 32 weeks) or with IUGR at the age of 19 years [18]. Among the preterm born children, 45.9% had prehypertension and 10.5% hypertension stage 1 or 2. In the group of IUGR, 37.6% had prehypertension and 8.8% hypertension stage 1 or 2 [18]. Mu performed a meta-analysis confirming that LBW increased the risk of developing hypertension by 20% [19]. Edvardsson has presented an extensive review on the inverse correlation between birth weight and blood pressure [20].

## Oligonephropathy

During normal development, nephrons are formed until 36 weeks of gestation, after which no new nephrons develop. At term birth, each kidney holds approximately 900,000 nephrons but a wide range in numbers has been described [21]. After the age of 60 years, there is a gradual loss of 6000–6500 functional nephrons each year [22]. The corresponding decrease in glomerular filtration rate (GFR) is proportionally smaller due to the compensatory mechanism of hyperfiltration by the remaining nephrons. Brenner and colleagues first described this hypothesis of glomerular hyperfiltration [3]. Compared with the natural decline in nephron number, a reduced nephron endowment, e.g., due to a solitary kidney or hypoplasia of the kidney, leads to compensatory hyperfiltration early in life. Glomerular hyperfiltration creates a higher capillary perfusion pressure leading to glomerular hypertension and glomerular hypertrophy with an expansion of the glomerular basal membrane (GBM). To cover the increased GBM surface, the length of podocyte foot processes increases and cell bodies stretch and thin. Since podocytes are unable to proliferate and transformation of glomerular parietal epithelial cells into podocytes is limited, hypertrophy of the existing podocytes is the only way they can adapt to the increase in GBM surface [23]. However the podocytes’ capacity to increase in size is lower than that of the GBM, which results in suboptimal coverage of the GBM with subsequent albuminuria. Together with the increased ultrafiltrate flow causing shear stress directly on the podocytes resulting in podocyte detachment, this leads to extensive denudation of the GBM, adherence of the capillary wall to Bowman’s capsule, glomerular sclerosis, and consequent loss of that nephron and its function. Progressive loss of functional nephrons eventually leads to CKD [23].

### Prematurity

The magnitude of reduction in nephron numbers upon preterm birth is dependent on the timing of delivery. Nephron formation starts at 15 weeks of gestation, with an exponential increase in nephrons in the third trimester, leading to a plateau around 36 weeks of gestation [24]. Before that time of cessation of nephrogenesis, nephron number is strongly correlated with gestational age [24] and therefore preterm birth results in a reduced nephron endowment. Rodriguez confirmed this theory in a human population [25]. Stelloh and colleagues also confirmed this correlation in a preterm mouse model [26].

There are several human studies showing that glomerulogenesis can continue after preterm birth [27]. Nephrons form in a centrifugal manner with the nephrogenic zone at the cortex acting as the location of nephron formation. After preterm birth, the radial glomerular generations, and maturational stages of glomeruli increase, and the nephrogenic zone decreases [28]. Glomerulogenesis after birth differs from intrauterine development as the maturation of glomeruli is accelerated, and more abnormal glomeruli are formed [25, 28]. To exclude potential confounding factors often encountered in human studies, animal models can be used. Gubhaju used a preterm baboon model (equivalent to 27 weeks of gestation in humans) to study the effect on nephrogenesis both at birth and 21 days postnatally. As in the human data, they showed that glomerulogenesis continues after birth with an increase of abnormal glomeruli from 1.3% at birth to 18.3% at 21 days [29]. Even though there is still some ongoing glomerulogenesis after birth, preterm born neonates do not reach the same number of nephrons as term neonates [29].

According to the hyperfiltration theory, in oligonephropathy, a reduced nephron number leads to hyperfiltration of the remaining glomeruli, resulting in glomerular hypertrophy with secondary damage to the GBM and podocytes, leading to proteinuria, glomerular sclerosis, and eventually kidney failure. Both Sutherland and Rodriguez found an increased renal corpuscle cross-sectional area in the first couple of months after birth as a morphological indication of glomerular hypertrophy [25, 28]. Brennen et al. visualized the nephrogenesis after preterm birth by ultrasound measurement of the total renal parenchymal thickness (TRP) [30]. In preterm neonates < 32 weeks of gestation, the TRP increases after birth, though it never reaches the thickness found in term controls. On the other hand, the parenchymal thickness to kidney volume ratio is increased. Authors therefore conclude that the reduced TRP is a radiological measurement of the impaired nephrogenesis after preterm birth, and the increased parenchymal thickness to kidney volume ratio is a visualization of glomerular hyperfiltration [30].

### Intrauterine growth restriction

IUGR is strongly correlated with reduced nephron endowment [31, 32]. However, IUGR is a heterogeneous group of patients due to the diverse etiologies, such as nutrient restriction, reduced uteroplacental blood flow and maternal preeclampsia [33]. Moreover, the lack of a clear definition of IUGR complicates research in this area. Often birth weight is used as a coarse marker for IUGR. However, LBW can be caused by IUGR, as well as prematurity or both, and early gestational IUGR with catch up growth does not necessarily result in LBW [7]. Even though differentiating the effect of each individual factor is difficult, research consistently shows that IUGR leads to reduced nephron number and kidney volume. Manalich provided human data comparing kidneys of full-term IUGR neonates (GA ≥ 36 weeks) with kidneys of full-term normal birth weight neonates and showed that IUGR had a 20% reduction in nephron number, with enlarged glomeruli [34]. A review by Schreuder et al. provides an overview of the animal data on IUGR and nephron number [32]. IUGR induced by either placenta embolization, surgical reduction of placental blood flow, steroid use, or maternal deprivation of nutreitens (either of total intake or of specific components such as protein, vitamin A, sodium, or iron) all lead to a reducation in nephron endowment [32].

Timing of IUGR during organogenesis is associated with risk profiles later in life, as shown by Roseboom and colleagues using the Dutch Famine Cohort [35]. Pregnant mothers experienced a period of severe acute food scarcity in the winter of 1944–1945. Follow-up data of the children subsequently born offer a unique insight into the effects of maternal starvation during a specific period of gestation. They proved that nutrient restriction during early gestation was linked to an atherogenic lipid profile and increased risk of coronary heart disease, while late gestational nutrient restriction was linked to glucose intolerance in adulthood [35]. Regarding the effect of timing of IUGR on nephron endowment, studies show that it influences the magnitude of nephron reduction. Early gestation IUGR in a rat model by unilateral umbilical vessel ligation has shown 21% reduction in nephron endowment [36]. In a sheep model where pregnant ewes were fed a 50% nutrient restricted diet, 11% nephron reduction was seen [37]. On the other hand, late gestation IUGR has proven to result in a higher reduction of nephrons. Postmortem analysis by Hinchliffe et al. demonstrated a 65% lower nephron number [38]. An animal model using rats that were subjected to a nutrient restriction in the last phase of nephrogenesis showed a 35% reduction in nephrons [39]. It can be argued that the magnitude of nephron reduction is not solely based on timing but also depends on etiology. However, to date, there is insufficient evidence to support this theory.

Glomerular maturation in neonates born after IUGR with ongoing nephrogenesis may be altered more severely, compared with prematurity alone. Rodriguez reported human autopsy data of neonates after preterm birth with birth weight < 1000 g. Overall, the authors concluded there is no postnatal ongoing glomerular maturation beyond 40 days after birth as ongoing subcortical nephrogenesis and S-shaped glomeruli were only found in the preterm group autopsied within 40 days after birth [25]. However, while this cessation of postnatal nephrogenesis after 40 days could be attributed to postnatal age, this could also be explained by the difference in intrauterine growth, as the neonates surviving over 40 days were predominantly SGA. On a pathophysiological level, this could mean that IUGR reduces the kidneys’ capability for postnatal nephrogenesis and could therefore lead to an even more significant reduction in functional nephron number.

### Chorioamnionitis

Chorioamnionitis is a bacterial intrauterine infection characterized by acute granulocyte infiltration in the fetal-maternal or fetal tissues. Chorioamnionitis is the most frequent cause of preterm delivery. Up to 50% of preterm birth is associated with chorioamnionitis [40], with reported incidences up to 94% in births between 21 and 24 weeks gestation [41]. Importantly, perinatal infection/inflammation, even if bacteria are not isolated, may lead to the fetal inflammatory response syndrome (FIRS) [42]. Chorioamnionitis and FIRS disrupt normal organ development by reducing growth and maturation as is shown in brain, lung and gut research [43–45]. It is therefore likely that inflammation also has a disruptive effect on the developing kidney.

Research data on the effect of intrauterine inflammation on nephron development is currently only available from pre-clinical models, in which intra-amniotic (IA) lipopolysaccharide (LPS) delivery induces chorioamnionitis and FIRS. The first published study regarding nephron development shows that a single IA high-dose injection of LPS given at the equivalent of 33 weeks human gestation leads to a 23% reduction in nephron number and increased renal corpuscle volume in lambs [46]. On the other hand, chronic LPS administration at a lower dose between the equivalents of 30 to 36 weeks human gestation did not lead to a reduction in nephron number [47]. Therefore, these combined findings suggest that the extent of infection and its timing (acute vs. chronic) determine the impact of chorioamnionitis on kidney development. However, the mechanisms by which nephron development is affected, for example, whether it affects the normal developmental cascade of nephrons in the nephrogenic zone or whether local or systemic inflammation with or without hypoperfusion of the kidney directly damages the maturing nephrons, remain elusive.

## Podocytopathy

Podocytes form an important part of the capillary filter in the glomerulus and are important in selective resistance to passage of larger molecules [48]. The cell-cell adhesions create small pores, slit diaphragms, which form a mechanical resistance. The negative charge of the cells (partly) prevents small negatively charged molecules, like albumin, passing though the filter. Simultaneous with glomerular development, podocytes develop, and both podocyte maturation and podocyte number are important factors when regarding this filtration function.

Podocyte development starts in the comma-shaped stage of glomerular development with mesenchymal cells differentiating into primitive podocytes [49]. In the S-shaped stage, the podocyte layer is made up of simple columnar-shaped podocytes connected with tight and adherence junctions. In the following capillary loop stage, primitive glomerular capillaries invaginate into the epithelial layer formed by the podocytes and GBM increasing the surface covered by the podocytes. In this stage, podocytes start to form primitive foot processes. In the subsequent maturing glomerular stage, foot processes continue to form and interdigitate with neighboring podocytes. It is not until the end of this stage and into the mature stage that tight and adherence junctions are replaced by slit diaphragms [49].

As podocytes are highly differentiated cells with limited capacity for replacement, their number per glomerulus is mainly determined during development. Kikuchi showed that the podocyte number increases until the capillary loop stage, after which the number remains static until full development [50]. However, with ongoing podocyte development, their volume increases and because of glomerular volume increase, the podocyte density decreases. Throughout life, the podocyte number gradually decreases. This process is accelerated in diseases where podocyte injury leads to increased podocyte loss, such as diabetic nephropathy, and eventually results in glomerulosclerosis [48].

### IUGR and prematurity and its impact on podocyte development

The effect of IUGR on podocytes in an animal model of protein restriction has been studied by Menendez-Castro and Chen [51, 52]. In both models, IUGR leads to a significant increase in blood pressure and proteinuria. On a cellular level, IUGR reduced podocyte maturation, as based on reduced expression of nephrin and synaptopodin, and increased podocyte damage, as shown by an increase in desmin and foot process effacement. Neither of these two studies quantified the number of podocytes [51, 52]. There is no data regarding the effect of chorioamnionitis on podocyte development.

In human cohort studies of patients with nephrotic syndrome, IUGR and prematurity have been found to be a risk factor for the development and aggravated course of nephrotic syndrome (NS). Several studies have shown that LBW and IUGR are associated with a steroid-dependent and steroid-resistant course of nephrotic syndrome [53, 54]. Compared with normal birth weight, patients with LBW more often require immunosuppressive agents, have more relapses, and require a higher cumulative dose of steroids [55]. A study by Ikezumi et al. in patients with NS showed that those with LBW were more likely to have FSGS on biopsy and had a 33% reduction in podocyte number [56]. Also, glomeruli were enlarged and glomerular density was lower in biopsies with FSGS of children with LBW [57]. However, since the birth weights were appropriate for gestational age, this effect is thought to be due to prematurity.

Additional to the effect IUGR has on the course of NS, there is evidence that it also aggravates the course of other glomerular diseases that potentially progress to glomerulosclerosis. Plank et al. studied two animal models of IUGR, by uterine artery ligation and maternal protein restriction, and induced glomerulonephritis [58, 59]. In both models, IUGR was associated with a higher degree of glomerulosclerosis expressed by the increased deposition of collagen I and IV in the glomeruli. Whether this increased susceptibility to develop glomerulosclerosis is also present after preterm birth or chorioamnionitis, is currently still unknown.

## Vascular development

Glomerular vasculogenesis is regulated through the renin-angiotensin system (RAS) and vascular endothelial growth factor (VEGF) cascade. Vascular development within the glomerulus starts in the S-shaped phase with podocytes excreting VEGF-A leading to the infiltration of endothelial cells, which subsequently excrete PDGF-β causing the migration of mesangial cells into the glomerulus. Mesangial cells are essential for the vascular loop formation and therefore increase the glomerular surface area [60]. On the other hand, the RAS, mainly via angiotensin II and the AT1 receptor (AT1R), both has a direct growth-enhancing effect and stimulates other growth factors in the developing kidney [61]. Inhibition of RAS during nephrogenesis is known to result in tubular dysgenesis, a reduced nephron endowment, and impaired capillary lumen formation and is strongly correlated to adult hypertension [61]. The interplay between RAS and the VEGF cascade in nephrogenesis is not yet fully understood.

The renal microvascular development encompasses not only glomerular vasculogenesis but also the development of peritubular capillaries. The development of these peritubular capillaries is also regulated by VEGF and RAS [61]. Studies have shown that peritubular capillary rarefaction is associated with the development of CKD [62] and an association with perinatal stress could be hypothesized. However, to date, literature on the effect of perinatal stress on peritubular capillaries is scarce. Therefore, this section of the review will be restricted to the impact of prematurity, IUGR, and chorioamnionitis on glomerular vasculogenesis.

### Vasculogenesis in prematurity

Sutherland and Staub both used preterm sheep models with sheep born at 130 days of gestation (comparable to 35 weeks gestation in humans) [63, 64]. Both studies found normal glomerular numbers, which can be explained by the gestational age by which nephrogenesis is complete. They did however find differences in vascular development. Sutherland described preterm animals to have a reduced glomerular capillary length, surface area, and total kidney filtration surface area [63]. Staub compared the effect of 3 and 21 days of mechanical ventilation [64]. After preterm birth, glomerular capillarization increased. However, mechanical ventilation inhibits this expected growth and therefore decreases the glomerular capillary development even further [64]. In a baboon model of prematurity, Guhbaju found an increase in abnormal glomeruli, defined by a cystic Bowman’s space and shrunken glomerular tuft [29]. The abnormal glomeruli were located in the outer kidney cortex and exhibited reduced CD-31 immunostaining, indicating disrupted development of vascular structures in the glomerular tuft, but expressed the same amount of VEGF as morphologically normal glomeruli. The authors therefore argue that the disrupted vascular development in prematurity is not caused by an absence of glomerular VEGF in the normal signaling pathway [29].

### IUGR affects RAS during vascular development

The correlation between IUGR and glomerulovascular development has been studied in animal models. Baserga is the only one to report on the VEGF cascade and found a downregulation of VEGF in the kidney during nephrogenesis in a rat model with growth restriction secondary to uteroplacental insufficiency [65]. At 21 days after birth, there was no difference in mean glomerular area, the only marker reporting on the quantity of vascular development. With progressing age, the mean glomerular area enlarged and the male rats had an increased urinary protein/creatinine ratio, which is described to be consistent with hyperfiltration [65]. The effect of IUGR on RAS seems to be related to the timing during gestation. Maternal protein restriction in rats, a species in which nephrogenesis continues until 7–10 days after birth and therefore represents early gestation IUGR, leads to downregulation of intrarenal RAS [66]. However, in an ovine model late gestation IUGR showed no difference in systemic or intrarenal RAS between the IUGR and normal birth weight group [67]. Therefore, the effect of IUGR on vascular development seems to predominate in early gestation. However, there is no data specifically correlating the downregulation of RAS to glomerular vascular development.

### Chorioamnionitis and glomerular capillaries

Compared with the aforementioned studies on prematurity and IUGR and their effect on glomerular vasculogenesis, research involving chorioamnionitis has focused on the postnatal effect on RAS and the subsequent development of hypertension—one of the known long-term consequences of perinatal stress. Ding et al. and Hao et al. showed in rodent models that chorioamnionitis increased the intrarenal expression of renin and angiotensin II in the weeks after birth without elevated levels of plasma renin activity or angiotensin II [68, 69]. Hypertension developed simultaneously with the rise in intrarenal RAS. This development of hypertension with increased intrarenal RAS and with low plasma renin is consistent with the salt-sensitivity hypertension model of Franco et al. in which hypertension is based on the inappropriate reabsorption of salt and water [70]. The effect of chorioamnionitis on RAS could therefore (partially) explain the development of hypertension associated with perinatal stress.

Another effect of chorioamnionitis on the glomerular capillaries is the correlation between chorioamnionitis and glomerulovascular damage rendering the glomeruli increasingly susceptible for glomerulosclerosis [71]. Kidneys exposed to LPS during development show more apoptosis at birth, mainly originating from glomerular endothelial cells [69]. Guo et al. showed that in adulthood, the glomeruli show increase in mesangial matrix, deposition of collagen 1 and 3 and upregulation of α-SMA in the interstitium [71]. These markers are consistent with fibrosis development and can explain the glomerulosclerosis seen in some glomeruli in this model after 68 weeks. The authors did not investigate glomerular numbers and hypothesize that the increased susceptibility to glomerular damage later in life is due to increased angiotensin II, which has chemo-attractant properties for macrophages and lymphocytes [71].

## Conclusion

The predisposition to develop CKD after preterm birth and/or perinatal stress, such as IUGR and chorioamnionitis, has long been acknowledged. Since Brenner’s hyperfiltration theory supported the correlation between oligonephropathy and proteinuria, hypertension, and CKD, initial research mainly focused on the correlation between prematurity, perinatal stress, and oligonephropathy. However, ongoing research has shown that the described predisposition for CKD involves pathways beyond oligonephropathy. Research has shown that IUGR is strongly associated with the risk of proteinuria [5] and podocyte development is negatively influenced by perinatal stress. Though there is no data on the quantity of the podocytes per glomerulus, there is evidence of reduced podocyte maturation and podocyte damage after IUGR [51, 52]. Moreover, IUGR negatively influences the course of NS [53, 54]. More recently, research has been conducted correlating prematurity and perinatal stress with glomerular vasculogenesis and postnatal glomerular damage. Though preterm birth is associated with reduced glomerulovascular development, this is not yet proven for IUGR or chorioamnionitis. However, it is known that the latter two risk factors give rise to increased intrarenal RAS and subsequent hypertension postnatally [65, 66]. Finally, in chorioamnionitis, glomerular damage predisposes to glomerulosclerosis [71].

All three aforementioned factors individually increase the risk of kidney disease later in life (Fig. 1), and these effects might be even stronger when these risk factors act synergistically. Where LBW, IUGR, and preterm birth increase the risk of CKD 5 by 50–70%, the combination of all three increases it threefold [10]. Therefore, all individuals born with at least one of these risk factors should be followed up in order to screen for kidney involvement in the early stages. This provides the opportunity to start treatment for hypertension and albuminuria, for example, with RAS inhibition, aiming to slow down the progression of kidney function deterioration. Moreover, the expected aggravated course of NS justifies earlier escalation of immunosuppressive treatment. As pointed out in this review, there are still gaps in knowledge regarding the pathophysiology of kidney disease after prematurity and perinatal stress. Further research is required to address these gaps and provide more targeted treatment options to reduce the long-term consequences on the kidney.
